# Extra Limites
*The Extra Limites department of this review [the regular limits are fourteen sheets, or 224 pages] was opened for the accommodation of public bodies or private individuals, as a medium through which they might publish, so as to secure a most extensive circulation, at the bare expense of the paper and type. This expense is not more than 9*s*. 6*d*. per page, the number of copies struck off, being at present 1500. It is requested that all papers for this department may be transmitted *(free of expence)* to the publishers of the Journal, at least sixteen days before publication day, the Journal being always closed on the 20th of the preceding month, to give time for binding and doing up the work.N. B. It is the intention of the Editor to defray a sheet (16 pages) of the Extra Limites department in each succeeding number, at his own expense, for the purpose of giving insertion to original papers of *superior value*. This will be a bonus to subscribers, and a mark of distinction on the papers themselves, which will come under the head of "Honorary Contributions." It is hardly necessary to state that these papers must be very select, succinct, and practical. They must be transmitted, free of expense, to the Editor, who will either publish them himself, or, if he has not room, transmit them back to the authors, or, if agreeable, will convey them to some cotemporary print, and afterwards notice them in the Periscope.


**Published:** 1822-09-01

**Authors:** 


### XIII.

### EXTRA LIMITES.*

### I.

*Observations on the Medical Use of the Oleum Terebinthinæ Rectificatum.* By WILLIAM MONEY, Surgeon to the Asylum for the Recovery of Health; to the Royal Metropolitan Infirmary for Sick Children; formerly House Surgeon to the General Hospital at Northampton; Member of the Medico-Chirurgical Society of London, &c. &c.

THE following observations on the use of the oil of turpentine as a remedy of great power in the cure of several more or less severe diseases will, I think, be interesting as well as somewhat novel, although much has of late been published on the same subject. These observations are selected from clinical notes made at the Northampton Hospital, between the years 1811 and 1816, comprising histories of a very extensive series of cases.

I shall not enter into a long detail of cases, but will state at once the more important facts they embrace, and the inferences that may be drawn from them. I have witnessed the administration of this oil in all the intermediate doses from fifteen drops taken two or three times a day, on to the large quantity of four fluid ounces at one dose. It has been

* The EXTRA LIMITES department of this review [the regular limits are fourteen sheets, or 224 pages] was opened for the accommodation of public bodies or private individuals, as a medium through which they might publish, so as to secure a most extensive circulation, at the bare expense of the paper and type. This expense is not more than 9*s*. 6*d*. per page, the number of copies struck off, being at present 1500. It is requested that all papers for this department may be transmitted *(free of expence)* to the publishers of the Journal, at least sixteen days before publication day, the Journal being always closed on the 20th of the preceding month, to give time for binding and doing up the work.

N. B. It is the intention of the Editor to defray a sheet (16 pages) of the EXTRA LIMITES department in each succeeding number, at his own expense, for the purpose of giving insertion to original papers of *superior value*. This will be a bonus to subscribers, and a mark of distinction on the papers themselves, which will come under the head of "HONORARY CONTRIBUTIONS." It is hardly necessary to state that these papers must be very select, succinct, and practical. They must be transmitted, free of expense, to the Editor, who will either publish them himself, or, if he has not room, transmit them back to the authors, or, if agreeable, will convey them to some cotemporary print, and afterwards notice them in the Periscope.

given in a variety of diseases, and in all, excepting one, was productive of great and obvious advantages.

By the small doses, as half a dram and less, taken repeatedly in the day, obstinate chronic rheumatic pains have been removed.

By doses of one dram to two drams, twice, thrice, or four times a day, cures have been accomplished in adults, labouring for two years under epileptic fits.

In doses of six drams every other morning, and continued for three weeks, children at the age of twelve years have been roused from a cloudiness of intellect bordering upon idiotism of the melancholic kind.

As a vermifuge, it has been given to the extent of one, two, three, and four ounces for a dose, the patient fasting; and its beneficial operation has not been confined to the tape-worm; but all the species of worms have been alike expelled.

In cases where several anomalous symptoms existed, such as pain in the region of the stomach, distention of the abdomen, irregular bowels, slight but irregular paroxysms of fever, wandering pains, pulse full, and disinclination to labor, without any emaciation; in these cases, in doses of two, three or four ounces taken fasting, in the morning, it has produced the speediest and best effects.

I shall now mention, in a cursory manner, a few cases.

*Case 1st.* A delicate female, aged 17, who having been long troubled with the tape-worm, had taken many medicines for it. She often passed fragments of the worm, and upon her admission in 1811, was much emaciated. Nearly the whole class of vermifuge medicines was tried, but without any success. The ol. terebinth. was then administered in the dose of one ounce, taken in a morning fasting, by itself, with an injunction to avoid food for two hours. She experienced slight heat in the fauces, with increased sensation of warmth at the stomach; and in about an hour and a half she had two copious evacuations, in which there were many links of the worm. In three days she repeated the medicine, in the quantity of two ounces. Four days from the second dose, she took the two ounces again at six o'clock, A. M. and another ounce at seven; from this she had four copious evacuations, accompanied with large quantities of the worm. On the 7th morning from the third dose, she took two ounces at six, one at seven, and another at eight o'clock, A. M. and was purged four times, but without passing any worm. This dose produced flushings in the face, giddiness, and a slight sense of heat in passing the urine, but these symptoms subsided after each evacuation. From this period her health rapidly improved, and she was shortly discharged, cured.

*Case* 2. A man, aged 30, having the tape-worm, took the oil as administered in the subject last mentioned, and was cured; but in this instance no symptom was produced further than the evacuations with the expulsion of the worm.

*Case* 3. A man, aged 48, complaining of the anomalous symptoms above designated. The dose of the medicine administered here was increased until he took at one draught four fluid ounces; this was at seven o'clock in the morning. He was soon purged, and experienced no ill effects until four o'clock in the afternoon, when slight strangury, pain in the head with giddiness, and acceleration of the pulse, came on. Barley water, with some castor oil, removed these symptoms in a few hours, and his other complaints were cured.

*Case* 4. Sunday, January 14th, 1816.—A stout man complaining of anomalous symptoms, took this morning, at seven o'clock, one ounce and a half for his first dose. In about three quarters of an hour, he felt an inclination to have a stool, and in the attempt to get out of bed for the purpose of going to the closet, he fell backwards, and was seized with spasms; this attack was quickly succeeded by a severe epileptic fit, which continued for thirty minutes, and left him senseless. In the course of the day he had three more fits, as severe as the first, and during the ensuing night, and the early part of the next day, he suffered constant spasms and twitchings. His countenance was sometimes much flushed, at others pale; his breathing occasionally sonorous and laborious, but generally quick; his pulse was oppressed and full, and sometimes intermitting; the heat of his skin was also irregular. His belly was hard and distended. He had involuntarily voided, during the fits, a small quantity of urine; but in consequence of the distention of the lower part of the belly, the catheter was introduced, and two ounces only of very pale urine, smelling slightly of the turpentine, were passed. No evacuation, per anum, took place until the middle of the following day, and he was in a state perfectly senseless until that time.

At one period, I considered the case as likely to prove fatal, and am convinced that such would have been the result, had not the most attentive and active treatment been pursued. When he became purged, the amendment first appeared. On Tuesday the spasms had entirely ceased, his urine was now bloody and thick, and voided not without uneasiness; he suffered pain in the region of the kidnies and constant pain also in the head. His bed had the odour of a strong bed of violets. Mild emulsions, diluents, and purgatives were now employed. On Thursday the urine became clear, and no distress remained except the pain in the head. On the ensuing Monday a seton was made in the nape of the neck, slight aperients were administered, and the warm bath directed to be used at a low degree of temperature. With this treatment, slightly varied, he was shortly discharged, cured.

*Case* 5. A boy, nine years old, had, when at the age of five years, a strong epileptic fit. He had another less severe in three weeks; this was followed, after short intervals, by others, differing in their degree of violence. For the last two years he has been in a state of idiotism, and has not passed one night during the last thirteen months without a fit, and he has frequently had them in the day. One night he had twelve. His urine is passed involuntarily; but he has the power of retaining his stools, which are very irregular, and his bowels are generally costive.

This boy commenced the use of the ol. terebinth. rectif. on the 15th of February, and left it off on the 7th of April; and during the period here indicated, he took of it two pints and ten ounces. It was administered at first in the doses of half a dram four times in the day, and the dose was at length increased to three drams, which was not at any time exceeded, whilst it was continued for nine successive days.

From the 15th of February to the 1st of March he had nineteen fits, which occurred after longer intervals, were less severe, and of shorter duration than heretofore. From the 1st of March to the 28th of the same month, he had no fit. On the 29th he had a very weak fit, which lasted about five minutes. From the 29th to the 16th April he had not a recurrence of the paroxysm. On the 16th of April he left the hospital at the request of his parents.

The changes that resulted from this treatment was as follows:—

1st. The pulse at the commencement was irregular, small, and quick. On the ninth day of the treatment it became regular, but continued small and quick. This regularity was disturbed for a few days; but ultimately the pulse was free, soft, and regular.

2dly. The bowels were slightly purged, and regular. No worms were voided.

3dly. The incontinence of urine was entirely obviated.

4thly. The fits, when they occurred, were less severe, and took place only after longer intervals, and the patient had but one fit during the subsequent six weeks, and that lasted only four or five minutes. And,

5thly. At first his intellects were so dull, that he was perfectly idiotic; when he had taken the medicine about three weeks, he became noisy, mischievous, and very troublesome, and occasionally would point to his head as if in pain there. In this state of cerebral excitement, he continued for a week. He then improved, and the amendment continued progressive, showing itself by a remarkable docility of disposition, and by a desire to read and to write.

The length of time during which this boy had laboured under his malady having been so considerable, the permanency of his improvement may be very doubtful, and I cannot, by any means, consider him as cured. But, so obvious and striking was the amendment in this case, it excited in me sensations so gratifying, peculiar, and inexpressible, that although the cure may be imperfect, I am still desirous of relating it in this paper, in order that other practitioners may give it a trial in similar, hopeless, and melancholy disorders.

In continuing to relate additional cases, I should only offer a tedious repetition of such as have been favourable; for I have nothing to record of a contrary kind, beyond those I have noticed in a former part of this memoir. In making this selection, I have had principally in view such cases as have reference to the administration of the oil in large and frequent doses; because in small doses it has, for many years, received the sanction and recommendation of estimable authorities.

This recommendation, however, was accompanied with a caution to limit with accuracy the dose to a few drops; and in order to enforce this caution, cases have been detailed, in which the most violent, and, in some instances, fatal effects, were the consequences of exceeding the prescribed dose. Where death or severe disease has been the consequence, I consider that we are *either* to attribute it to some idiosyncrasy of the patient; or to the dose having been so intermediate between the small and the large quantity as not to have been sufficient to excite that energetic action on the stomach and alimentary canal, which appears from recent experience to be its more immediate operation. If we should suffer the first of these causes, namely, some idiosyncrasy, to deter us from the use of this or any other active medicine it seems probable that, at no very remote period, we should relinquish some of the most important remedies that are now relied upon in the treatment of diseases; the use of which, have been founded, for the most part, on physiological reasonings and observed facts. Cases corroborative of this opinion are numerous and frequent; but I will nevertheless mention two, which regard the use of that powerful medicine, mercury.

A man, aged thirty-five, labouring under certain complaints for which mercury was considered necessary, commenced by direction to rub on his legs and thighs one dram of the unguentum hydrargyri fortius on a Tuesday night. This was repeated on the Wednesday, Thursday, and Friday nights, when a severe ptyalismus supervened, his face swelling much, accompanied with a copious discharge of saliva. Upon this the friction was omitted. He was debilitated, and his bowels severely affected with diarrhœa; this was at first slightly encouraged, and then moderately checked. Early in the morning of the following Tuesday, great prostration of strength suddenly occurred, his pupils were insensible and contracted, his extremities cold and livid, he passed into a comatose state, and died on the Wednesday night. It appears that the quantity of ointment used was only four drams; and this quantity produced consequences rapidly fatal.

At the time the last-mentioned patient was under treatment, a female, aged fifteen, had used, by means of friction, in the course of four weeks, nearly four ounces of the ointment prepared in the same manner, and had taken, in small doses, during this period, as well as three weeks previously, several drams of the submuriate of mercury, without any apparent effects.

Are we then to abrogate the use of mercury in the treatment of diseases? Or, are we even to be more scrupulous than hitherto in the use of it, on account of such cases as those which I have related? Upon similar grounds I consider we are to decide upon the use of the oleum terebinthinæ rectificatum, when administered in large doses; although the results of the fourth case related in this paper, were unfavourable or deleterious.

In cases where any idiosyncrasy does not appear to exist, I should refer the deleterious consequences to the dose not having been large enough; for instance, forty or fifty drops given twice or three times a day, will often produce strangury and bloody urine, with the other ordinary concomitant symptoms, whilst in the same subject one, two, three, or four ounces, cause no such effect; but operate in the manner described in this memoir.

Experiments and observations, which I do not think it necessary to relate here, have led me to entertain an opinion that where the small doses have been given and followed with the violent symptoms remarked by the older authors, the oil being retained too long in the stomach, exerts an undue action on the nerves of this organ; and by sympathy or other relations, on the functions of the brain; and hence the respiration becomes disturbed, and the secretions are changed and vitiated, but more particularly that of the urine; and according to the extent of this preternatural action or excitement, we are to expect more or less of severity in the consequent symptoms. This I will briefly illustrate by an experiment. A man took early in the morning one dram of the oil; in about ten minutes, he began to experience a sense of heat at the stomach, which was followed by pain in the head, his pulse and respiration became quickened. He shortly afterwards voided a small quantity of urine, denoting the effects of turpentine. In the course of three hours he became easier. In the afternoon he had a slight return of these complaints, and the urine presented more powerfully the odour of the oil. At five o'clock in the afternoon he had a gentle purging evacuation; the abovementioned symptoms then began to subside, and soon totally disappeared. Four days afterwards he took another dram of the oil;—in about forty minutes he suffered the sense of heat at the stomach, and the pain in his head, as described in the last experiment; immediately he took one ounce and a half more; the symptoms did not increase in severity, he was quickly purged, and by noon he experienced no effects of the medicine. For these reasons I am of opinion that it is better to give the oil in doses of an ounce and a half or two ounces, repeating it at the intervals of an hour, according to circumstances, than to give, at one dose, the same quantity as was taken in Case 3 of this paper. Because, if the immediate effects were of the most aggravated kind, then, I conceive, a repetition of the medicine would be rapidly fatal; on the other hand, if symptoms were to follow similar to those which appeared subsequent to the experiment, then I should rely upon a repetition; knowing, from a number of other experiments, where this medicine, in small doses, operated in the way mentioned, that the most efficient remedy is, the immediate administration of a large dose, by which the energetic action of this oil is demonstrated.

I could bring forward additional facts and experiments; but I trust that I have here presented such a series of observations, as will be deemed sufficient to direct the attention of other practitioners to this remedy in a manner commensurate with its merits.

The author cannot allow this opportunity to pass without thanking the medical officers attached to the hospital during the period referred to in the course of this paper, for the kindness and indulgence he experienced from them collectively, but more particularly the superintendent, Dr. Kerr, whose attention was very much directed to the facts herein detailed. The author further begs to say that if the officers belonging to eleemosynary institutions will give the medicine a full trial, he is convinced that the funds of these charities will be thereby materially benefited.

*Hanover Street, Hanover Square.*

*London,* 1821.

### II.

*Annual Report of the General Committee of the Associated Apothecaries and Surgeon Apothecaries of England and Wales, received and adopted at the Annual General Meeting of the Association, held by Public Advertisement, at the Crown and Anchor Tavern, Strand, July 3d,* 1822.—*Joseph Hayes, Esq. President.*

Your committee, with all due respect, submits to the general meeting the following report of its proceedings during the past year, and of the present state of the Society.

From the formation of this Institution, the principal object which the association has had in view has been the advancement of the usefulness and respectability of the medical profession, by effectually restraining the uneducated and the ignorant from practising an art, which, above all others, requires patient study and close attention, investigation the most minute, knowledge the most varied, and application the most intense.

It was soon perceived that this end was attainable only through the medium of legislative interference. The necessary interposition was therefore respectfully sought for, and principally *through the exertions of this Society,* and to a certain extent obtained.

Had the Act of Parliament alluded to been efficient to the purposes required, little had been wanting to the public security from the misconduct of rash pretenders to medical skill, or to the well-being of the general practitioner; but while it is acknowledged by all who are competent to judge, that in the law referred to, ("commonly called the Apothecaries' Act,") there are ambiguities and defects, which can be remedied only by a new enactment, your Committee regrets that no favourable opportunity has offered of applying to Parliament for relief.

From such an application the Committee has been deterred by different considerations; the fate of the Surgeons' Bill, and even of the Apothecaries' Act, "curtailed of its fair proportions," during its progress through the Upper House, tended greatly to discourage such a step; nor did the state of the funds warrant an attempt which would certainly be expensive, and probably unsuccessful; a still more cogent objection existed in the paucity of information, yet prepared to be laid before Parliament, as to the abuses which prevail.

To collect proofs of mal-practices, and to acquire extensive intelligence of the various and complicated mischiefs which are perpetually springing from the gross ignorance and often unprincipled conduct of empirics, has been the constant aim of your Committee; for this purpose a correspondence has been carried on (through your secretary) with the most intelligent and respectable practitioners in different parts of the kingdom, and every accessible source of information appealed to;—the result has been conformable to the apprehensions of the Committee; and the public, as well as this Society, will soon be made acquainted with a frightful detail of evils, which unhappily, however deeply they are to be deplored, do not seem, at present, within the reach of any adequate remedy.

The state of midwifery, in particular, is deserving the most serious attention of the Legislature; exercised, as that art frequently is, by persons without the slightest pretension to anatomical or medical knowledge. It is painful to contemplate the miseries which are inflicted by the ignorance and violence of men who unblushingly profess an acquaintance with this branch of the profession, though they have but recently followed the plough, or laboured at the loom. Nevertheless, your Committee does not despair of future amelioration.

"Quicquid erit superanda omnis fortuna ferendo est."—*Virgil.*

When more numerous instances shall have been collected (and it cannot be doubted that they are every where to be found) and when these shall have been so arranged and embodied as to supply convincing proofs of the necessity of legislative measures, then will have arrived the proper time for an application to Parliament; we may then reasonably claim attention and expect redress.

The protection not merely of ordinary sufferers from mischievous and often fatal treatment, but of afflicted mothers and their helpless infants, cannot be deemed unworthy of the especial deliberations of an enlightened legislature.

Meanwhile the Committee recommends to every member of this extensive association to acquire and transmit information of glaring ignorance and misconduct, wherever they may be found, to the secretary; that it may be maturely reflected on, and, if necessary, forwarded to the Society of Apothecaries for ulterior consideration.

Since the last annual meeting another instance has been afforded of the utility of the Apothecaries Act by the successful prosecution of an individual not duly authorized to practise medicine; and that society has further declared itself ready to enforce the provisions of the Act against all who wantonly violate it, whenever conclusive evidence against the offenders can be obtained.

In collecting and forwarding such information, it is earnestly recommended that the greatest care be employed to avoid exaggeration or misstatement, that truth only be the basis of the communication:

rather "extenuating than setting down aught in malice;" and above all, that the energies of its members be in every possible mode directed not merely to the peculiar interests of the association, but to the advancement of science, the honour of the profession, and the public welfare.

Your Committee now gladly turns to another and more pleasing part of its duty, viz. to announce that the several resolutions, Nos. 15, 16, 17, 18, 19, 20, and 21, passed at the last general meeting, relative to the publication of a volume on medical topics, have been complied with; the zeal and industry of the profession have supplied several essays, which, while they reflect credit on their respective authors, will, it is confidently anticipated, confer additional estimation on the profession itself, both by encreasing the mass of practical information, and giving it a wider spread.

The printing of the volume alluded to, to be entitled "Medical Essays and Observations, published by the Associated Apothecaries and Surgeon Apothecaries of England and Wales," has been delayed by circumstances which it would be useless to specify; but the gentlemen to whom your Committee referred the arrangement of the papers, announce that the work is in the press, and that it now waits only for the receipt of the subscriptions necessary to secure the society from incurring any loss by a measure which the proposers intended for its welfare and advancement.

The mode of publication adopted is calculated to supply to every *subscribing* member an interesting volume, at a small price, and yet, by its sale, ultimately to sustain the diminishing funds of the Association.

At the commencement of an undertaking so novel, difficulties were to be expected—the avocations of some practitioners, and the timidity of others, tended to restrain the pens of gentlemen well qualified to detail interesting cases of disease, to investigate the causes and symptoms, and to suggest improved modes of treatment; but it may reasonably be hoped, that the members of the Association, in possession, as they must be, of much and valuable information, will hereafter feel less reluctance to write, and that from year to year our projected volume will increase both in magnitude and excellence.

Of the merits of the publication in question it might have been difficult for your committee to descant, were it not that practitioners of deserved celebrity have contributed to the stock of information; more than one physician, unconnected with this Society, except by an ardent desire to promote its welfare, and to benefit mankind, have obligingly furnished communications which would do honour to any existing work. It is not to be doubted that these will, on future occasions, be augmented in number, and serve materially to enhance the value of the book.

The state of the funds will be laid before you; it will there be perceived with regret, that notwithstanding the strictest economy on the part of your Committee, the expenditure far exceeds the regular income, and that in the event of again petitioning either House of

Parliament, another call must inevitably be made on all the members of the Society.

Actuated by no sinister motives—seeking no exclusive advantages, having in view less the *interests of the profession* than of the *community* at large, your Committee begs leave to express its decided opinion, that this Association is fairly entitled to the firm support, and zealous co-operation, not only of its own members, but of every enlightened medical practitioner, and to the countenance and good wishes of all feeling and virtuous men.

JOHN POWELL, *Secretary.*

1, *Keppel Street.*

### III.

*Remarkable Case of extensive Visceral Disease, attended with some peculiar circumstances.* By James Johnson, M. D.

Major Leonard, ætat. 65, residing in Crawford Street, had been complaining of stomach and biliary affections for ten years. About six years ago he found himself seized with dyspnœa and palpitation, on making any sudden exertion, or ascending an eminence. He noticed at this time also, that his pulse habitually intermitted. During the last four or five years, his stomach was much out of order, his stools irregular and of various colours, and his spirits sometimes depressed. About eighteen months ago he applied to Dr. Scott, who desired him to use the nitro-muriatic acid bath. From this application he derived the greatest benefit, and never afterwards left it quite off. It always relieved the uneasy sensations in his stomach, changed the colour of his motions, and improved his appetite. Last summer a considerable rash came out on his arms, thighs, and legs, producing great irritation in those parts; but during this eruption, his breathing was freer, his appetite better, his digestion easier, than for several years past. The eruption continued out more than two months, and towards its decline, about the middle of July last, he consulted me. He had then dyspnœa on going up stairs, his pulse was very irregular, feeble, and intermitting. His urine was getting scanty and lateritious, he had slight cough, and a trifling mucous expectoration.

On examining the chest it sounded badly on both sides, but especially on the right side. The action of the heart could be heard over a greater surface than in a healthy state, viz. over a diameter of six or eight inches, when the ear was applied to the chest. He was ordered the decoction of taraxacum, with one grain of blue pill and three of aloetic pill every night, regulating carefully his diet, and desiring him to live very quietly. Under this plan his urine became free, his motions better coloured, and his appetite keen. I only saw him about once in a month, till the 16th December, 1821, when he came to me, complaining that his appetite had left him rather suddenly; that he had sickness, and even vomiting, after eating; that his breathing was very uncomfortable after using any exertion; and that altogether he felt different from what he had done for a long time past. On examining the chest, it sounded worse than ever, and the action of the heart was exceedingly irregular. I requested a consultation with Dr. Baillie and Dr. Wilson Philip. In the mean time, he took soda water for drink, which entirely checked the sickness and vomiting, and they never afterwards returned. He was also cupped over the sternum, which relieved him a little. On the 20th December the consultation took place, and as it was considered by the physicians abovementioned that there was hydrothorax, some blue pill, and digitalis, were prescribed. On the 22d his breathing was rather worse, and blood appeared in the expectoration. He now expressed a wish to see Dr. Bree, who found him in a very low state, his pulse vacillating and feeble, and his extremities inclining to be cold. Dr. B. recommended (but without hope of more than temporary relief) cordials, and carbonate of iron. His expectoration, however, became decidedly sanguineous, and the breathing gradually more laborious. Yet he had no pain, no sickness at stomach, no fever;—in short, he complained of nothing but his breathing. The sanguineous expectoration continued the same, and the dyspnœa increased till he died, on the morning of January 1st, 1822.

*Minutes of Dissection, Jan.* 1*st*, 1822, *Eight o'Clock in the Evening. Present, Mr. North, of Seymour Place, Mr. Bartholomew, Mr. Harrison, and myself.**

*Head* perfectly sound, except a specula of bone in the falciform process of the dura mater.

*Thorax.* Ribs, all firmly ossified. About three pints of reddish serum in the right cavity of the pleura, and about half a pint in the left. The lung of the right side was as solid as liver, except a very small portion at the upper part of the chest. The air-cells were completely obliterated, and nothing but the bronchial tubes remained pervious. The lung exhibited precisely the appearance described by Laennec, as obtaining in violent cases of hæmoptysis, or, as he terms it, "*pulmonary apoplexy.*" The left lung was in the same state, but not to so great an extent. A considerable portion of the upper part of the lung was crepitous, and capable of the respiratory function. Specimens of the lungs were sent to Mr. Bell and Mr. Brookes.

The heart was nearly double its natural size—the cavities being dilated and the parietes thickened, constituting the hypertrophia of Laennec. The auriculo-ventricular opening on the left side was a

* I ventured to predict, before commencing the dissection, to Mr. North and the gentlemen present, that we should find effusion in the chest, hepatization of the lungs, and disease of the heart.

circle of bone—the mitral valve ossified.* No disease in the right cavities of the heart.

*Abdomen.* Liver diseased. It resembled, in appearance, a piece of granite—being variegated with innumerable whitish granulations, intermixed with the common structure. It was indurated, and very little vascular. The gall-bladder was full of bile, as black as ink. The stomach was very capacious, and about the middle of its larger curvature, a circular ulcer was found about the size of a farthing, penetrating the villous coat, and having hardened and raised edges. This ulcer was evidently of no recent formation; yet he had sickness and vomiting only for a few days—and during the last eight or ten days of his life, he had no sickness, pain, or other inconvenience in the stomach, on eating, drinking, or taking the hottest and most cordial medicines, which were sometimes given him to rouse the flagging circulation.

P. S. Major Leonard had not led an intemperate life; but played to a great extent, and lost large sums of money. These states of perturbation must have greatly disturbed the functions of the heart and stomach, and probably led to the organic diseases detailed.

J. J.

### IV.

### *To the Editor of the Medico-Chirurgical Review.*

Sir,

In a note inscribed in the course of the very copious and candid review of my late Treatise on Amaurosis, published in the March number of your highly valuable Journal, it is observed, (p. 788,) that "Lullier Winslow has somewhat anticipated Mr. Stevenson, relative to local plethora, being very generally concerned in the production of amaurosis."†

* At each contraction of the left ventricle, a portion of blood must have rushed back into the left auricle, from the imperfection of the intervening valve. Hence the feeble irregular pulse—and hence, perhaps, the dyspnœa originally, and hepatization of the lung subsequently, on account of the impeded return of blood from the lesser circulation.

† The reviewer of Mr. Stevenson's Work was wrong in attributing the doctrine in question to Lullier Winslow. This doctrine was distinctly laid down by Morgagni more than one hundred years ago; and he merely quotes it from Bonetus, Boerrhaave, &c. to confirm its truth, as the following passage will shew:—

"Porro sunt aliæ etiam causæ quæ nervos comprimant opticos, eoque amaurosim inducant, in sepulchreto commemoratæ, et medicis quaque observationibus comprobatæ, *nimia copia et turgentia sanguinis, arterias ac venas tumefacientis quæ intus extrave comitantur mollissimam eorum nervorum substantiam.* Qua explicatione recte utitur Boerrhaavius ad Ætii amaurosim exponendam, quæ in morbis capitis fervidissimis et post phrenititem sequitur." *Lib.* I. *de Morbis Capitis, Ep.* XIII. *Art.* 5.

Whilst I acknowledge the satisfaction I feel that a similarity of sentiment respecting a pathological point of much practical importance should have existed between a writer of great respectability and myself, I deem it incumbent upon me, in order to avoid the suspicion even of plagiarism which such a coincidence might excite, to declare that, until the quotation appeared in your Journal, I was wholly ignorant of the contents of the paper alluded to, the whole of which, on the *nature* and *cure* of the disease makes up only *three pages* of octavo print. To Lullier Winslow, therefore, I am not even indebted for the *hint* there thrown out, nor, I may add, to any other author for the theory I ventured to promulgate in regard to the proximate cause of one of the most frequent of the curable species of amaurosis. The theory in question, as is particularly stated in my Preface, first suggested itself to me more than twenty years ago, when engaged in extensive general practice, and has been gradually matured and moulded into its present shape, by the aid of much subsequent reflection, observation, and experience. And I flatter myself that an unbiassed comparison of the views, pathological facts, and mode of reasoning, adopted in my Treatise, with the remarks that are to be found in any preceding publication upon the same subject, particularly as regards those cases which are marked by great constitutional debility, and the absence of the usual indications of local congestion, will fairly establish my claim to originality, no less with respect to the doctrine, than the treatment of the disease. Such I am proud to state is the opinion avowed by a large number of the most distinguished of my professional brethren.

But in disclaiming any obligation to the authors who have treated of the same topic, I am sensible that an apology is due for the obvious marks of haste and want of precise arrangement visible throughout the Work. As an excuse for these defects, I would plead, on the one hand, the little leisure I could command from my professional occupations; and, on the other, the desire naturally felt, that the public might possess, with as little delay as possible, an ungarbled exposition of the peculiar opinions I had been led to form on the subject. The doctrines advocated in my Treatise have, during the short time that has elapsed since its publication, received much additional confirmation and support, as well from my own practice, as from the communications of my medical friends; and I hope to render a new edition of the work more worthy of their approbation, and that of the public.

I am, Sir, &c.

JOHN STEVENSON.

*Great Russell Street,*
*July* 1*st*, 1822.

---

To this cause Morgagni, not improperly perhaps, refers those temporary amauroses which sometimes take place in pregnant women, and disappear when the pressure of the gravid uterus is removed from the abdominal vessels. *Editor.*

